# Heparin Reduces Neuroinflammation and Transsynaptic Neuronal Apoptosis in a Model of Subarachnoid Hemorrhage

**DOI:** 10.1007/s12975-012-0166-9

**Published:** 2012-04-14

**Authors:** J. Marc Simard, Cigdem Tosun, Svetlana Ivanova, David B. Kurland, Caron Hong, Leanne Radecki, Carter Gisriel, Rupal Mehta, David Schreibman, Volodymyr Gerzanich

**Affiliations:** 1 Department of Neurosurgery, University of Maryland School of Medicine, 22 S. Greene St., Suite S12D, Baltimore, MD 21201-1595 USA; 2 Department of Pathology, University of Maryland School of Medicine, Baltimore, MD USA; 3 Department of Physiology, University of Maryland School of Medicine, Baltimore, MD USA; 4 Department of Anesthesiology, University of Maryland School of Medicine, Baltimore, MD USA

**Keywords:** Subarachnoid hemorrhage, Neuroinflammation, Microglia, Heparin, Transsynaptic apoptosis, Rat

## Abstract

Subarachnoid hemorrhage (SAH) can lead to disabling motor, cognitive, and neuropsychological abnormalities. Part of the secondary injury to cerebral tissues associated with SAH is attributable to the neuroinflammatory response induced by blood. Heparin is a pleiotropic compound that reduces inflammatory responses in conditions outside the central nervous system. Using a model of SAH devoid of global insult, we evaluated the effect of delayed intravenous (IV) infusion of heparin, at a dose that does not produce therapeutic anticoagulation, on neuroinflammation, myelin preservation, and apoptosis. Adult male rats underwent bilateral stereotactic injections of autologous blood (50 μL) into the subarachnoid space of the entorhinal cortex. The rats were implanted with mini-osmotic pumps that delivered either vehicle or unfractionated heparin (10 U/kg/h IV) beginning 12 h after SAH. No mechanical or hemorrhagic injury was observed in the hippocampus. In vehicle controls assessed at 48 h, SAH was associated with robust neuroinflammation in the adjacent cortex [neutrophils, activated phagocytic microglia, nuclear factor-kappa B, tumor necrosis factor-alpha, and interleukin-1beta] and neurodegeneration (Fluoro-Jade C staining and loss of NeuN). In the hippocampus, a muted neuroinflammatory response was indicated by Iba1-positive, ED1-negative microglia exhibiting an activated morphology. The perforant pathway showed Fluoro-Jade C staining and demyelination, and granule cells of the dentate gyrus had pyknotic nuclei, labeled with Fluoro-Jade C and showed upregulation of cleaved caspase-3, consistent with transsynaptic apoptosis. Administration of heparin significantly reduced neuroinflammation, demyelination, and transsynaptic apoptosis. We conclude that delayed IV infusion of low-dose unfractionated heparin may attenuate adverse neuroinflammatory effects of SAH.

## Introduction

Subarachnoid hemorrhage (SAH) comprises 5–7 % of all strokes [30, 38]. Acutely, 30 % or more of patients die from the initial hemorrhage or rehemorrhage [30, 38]. Patients who survive are at high risk for potentially severe neurological and neuropsychological complications that develop during the subsequent days and weeks, with well over half succumbing to cerebral vasospasm, stroke, hydrocephalus, or cognitive and neuropsychological deficits. Among the worst cases are those in which the hemorrhage involves the eloquent cortex, a situation frequently encountered with rupture of middle cerebral artery aneurysms [55, 56]. However, even among patients with mild hemorrhages (World Federation of Neurosurgical Societies grade 1) who do not suffer clinical or radiological vasospasm and who do not experience perioperative complications, long-term psychosocial and cognitive difficulties are still very common [6, 10].

Collectively, the various neurological and neuropsychological abnormalities brought on by SAH may be referred to as “delayed neurological deficits” (DNDs) [74]. Historically, the most devastating cause of DNDs was considered to be vasospasm leading to ischemia and stroke. Recently, a large clinical trial demonstrated that successful prevention of angiographic vasospasm by inhibition of endothelin signaling does not translate into improved clinical outcomes [48]. This landmark finding indicates that other mechanisms of secondary injury induced by blood, possibly unrelated to ischemia, play a crucial role in the development of DNDs [5, 8, 21, 31].

SAH has long been known to induce an inflammatory response in blood vessels and neuronal tissues [11, 17, 24, 29, 52, 61, 64, 72, 73, 77, 78, 84]. Blood incites a robust inflammatory response in the central nervous system that is attributable, in part, to oxidative stress [43]. Many conventional markers of inflammation are activated or upregulated by SAH, including leukocytes, microglia, immune complexes, complement, C-reactive protein, and numerous cytokines and chemokines [11, 22, 24, 37, 62, 73, 77]. Despite convincing evidence for the involvement of inflammation, block of conventional inflammatory/immune pathways using a variety of agents (glucocorticoids, lazaroids, iron chelators, cyclosporine, tacrolimus, and nonsteroidal anti-inflammatory drugs) has been of limited benefit in ameliorating DNDs associated with SAH, both in animal models and in humans [11, 72]. Nevertheless, recent studies examining nonconventional anti-inflammatory agents have shown promise [73, 77].

Heparin is a member of a family of polyanionic polysaccharides called glycosaminoglycans, composed of hexuronic acid and d-glucosamine residues joined by glycosidic linkages [9]. Although clinically utilized almost exclusively as an anticoagulant, heparin binding can interrupt numerous biological pathways [45]. Heparin is a pleiotropic drug that has long been recognized to have broad anti-inflammatory and immunomodulatory activities that are independent of its anticoagulant effect [18, 44, 45, 65, 80, 86]. On the extensive list of heparin-binding proteins are several important cytokines and all chemokines [18, 80, 86].

We recently reviewed the potential benefits of heparin as a pleiotropic, prophylactic agent in the context of SAH, showing how heparin can potentially antagonize most of the pathophysiological mechanisms known to be activated following SAH [74]. Here, we evaluated the effect of delayed intravenous (IV) infusion of heparin, at a dose that does not produce therapeutic anticoagulation [4, 42], in a model of SAH involving the entorhinal cortex, the part of the temporal lobe that projects directly to the hippocampus via the perforant pathway. In this model, SAH was associated with robust neuroinflammation in the adjacent cortex and white matter, with muted neuroinflammation in the hippocampus, with demyelination of the perforant pathway, and with transsynaptic apoptosis in the dentate gyrus. Here, we report that delayed IV infusion of low-dose heparin significantly reduced neuroinflammation, demyelination, and transsynaptic apoptosis.

## Methods

### Subjects and Experimental Series

All experimental procedures were approved by the University of Maryland Institutional Animal Care and Use Committee. Male Wistar rats (300–350 g; Harlan, Indianapolis, IN, USA) were used in this study, which was comprised of two experimental series: In series 1, eight rats underwent bilateral SAH (see below), three rats underwent unilateral SAH (right side only), and three rats underwent sham injury [same procedure but with injection of normal saline (NS) instead of blood]. They were euthanized at 48 h. These brains were used for pathological and histochemical characterization of the model, including assessment for infarction using 2,3,5-triphenyltetrazolium chloride (TTC) staining (three rats with bilateral SAH), assessment of tissue damage (Fluoro-Jade C staining) with saline vs. blood (three rats each with unilateral saline vs. blood), and evaluation of the hemorrhage location with hematoxylin and eosin (H&E) staining (five rats with bilateral SAH). In series 2, 16 rats underwent bilateral SAH and were implanted with mini-osmotic pumps that delivered either NS (8 rats) or heparin (8 rats; see below). These brains were used for further histochemical and immunohistochemical characterization of the model and to assess the effect of heparin treatment. In accordance with “good laboratory practice,” surgical procedures were conducted by investigators blinded to treatment.

### Rat Model of SAH

Anesthetized (ketamine 60 mg/kg and xylazine 7.5 mg/kg intraperitoneally) rats spontaneously breathed room air supplemented with oxygen to maintain 90 % < sO_2_ < 98 % by pulse oximetry. Temperature was maintained at 37°C using a heating pad regulated by a rectal temperature sensor (Harvard Apparatus, Holliston, MA, USA). Surgical procedures were carried out using aseptic techniques.

SAH was produced by injecting 50 μL of autologous blood under stereotactic guidance into the subarachnoid space of the entorhinal cortex, bilaterally. First, the tail artery was cannulated (PE-20 tubing) under microscopic visualization. The head was fixed in a stereotaxic apparatus (Stoelting Co., Wood Dale, IL, USA) and a midline scalp incision was made. One burr hole was placed at (bregma and midline as reference points) anterior–posterior, −7.7 mm; lateral, +5 mm; a second burr hole was placed at anterior–posterior, −7.7 mm; lateral, −5 mm. The dura at each burr hole was pierced sharply. Then, 600 μL of blood was aspirated from the tail artery; the last 125 μL of nonheparinized blood was aspirated into a syringe equipped with a 22-mm-long blunt, tapered-tip needle (Hamilton custom tapered syringe, 7732-01; Hamilton Co., Reno, NV, USA). The syringe was mounted in the stereotaxic apparatus and the tip coordinates were registered. Beginning at the burr hole on the right side, the needle was advanced to the *z*-coordinate of −7.0, then was advanced very slowly until the curvature of the posterior aspect of the middle fossa was reached, as indicated by the needle shifting slightly anteriorly [at the *z*-coordinate (mean ± SD), −8.3 ± 0.4 mm; range, −7.3 to −9.2 mm]. Use of a blunt, tapered-tip needle prevented the dura and bone from being pierced, allowing reliable access to the subarachnoid space. Without adjusting the depth, 50 μL of blood was injected over 5 min, after which 5 min was allowed to elapse before slowly withdrawing the needle. The procedure was repeated on the left side.

### Treatment

In rats that would be administered drug, IV access to the external jugular vein was obtained under microscopic visualization. A mini-osmotic pump (1.0 μL/h; Azlet 2001, Durect Corporation, Cupertino, CA, USA) fitted with a rat jugular vein catheter (#0007710, Alzet, Durect Corporation, Cupertino, CA, USA) was implanted. The catheter was filled sequentially with 11 μL of NS, 1 μL of air, and the remainder with the solution to be administered; the first 12 μL in the catheter provided a 12-h delay in the start of drug administration [75]. The pumps were filled either with NS or with heparin sodium (5,000 USP U/ml; APP Pharmaceuticals, LLC, Schaumburg, IL, USA) in NS (1 ml of heparin stock plus 0.43 ml NS) that delivered 10 U/kg/h. Therapeutic anticoagulation in the rat requires constant infusion of 75 U/kg/h of heparin [42].

### Histochemistry

After transcardiac perfusion/fixation with 10 % neutral buffered formalin, the brains were cryoprotected with 30 % sucrose. For routine histology, cryosections (10 μm) were stained with H&E and examined using light microscopy.

To assess myelin, cryosections (10 μm) were stained with Black Gold II according to manufacturer’s protocol (Black Gold II Myelin Staining Kit, AG105, Millipore, Temecula, CA, USA). Briefly, slides were first rehydrated and incubated in prewarmed 0.3 % Black Gold II solution at 60°C for 15 min. Sections were then rinsed twice, and then incubated in 1 % sodium thiosulfate solution for 3 min at 60°C. After several rinses with Milli-Q water, the sections were counterstained with cresyl violet to visualize nuclei, were coverslipped, and were examined using light microscopy.

To identify degenerating neurons and white matter tracts, cryosections (10 μm) were labeled with Fluoro-Jade C histofluorescent stain [70] according to the manufacturer’s protocol (Histo-Chem, Jefferson, AR, USA). Sections were coverslipped with nonpolar mounting medium (Cytoseal XYL, Richard-Allan Scientific, Kalamazoo, MI, USA) and were examined with an epifluorescence microscope (Nikon Eclipse 90i) under a fluorescein isothiocyanate filter, with care being taken to limit the time of exposure in order to reduce photobleaching.

### Immunohistochemistry

Cryosections (10 μm) were first blocked (5 % goat serum, Sigma + 0.2 % Triton X-100 for 1 h at room temperature) and then incubated overnight at 4°C with the following primary antibodies: rabbit anti-ionized calcium-binding adapter molecule 1 (Iba1) (1:1,000; Wako Chemicals, Richmond, VA, USA); mouse anti-ED-1 (1:200; MAB1435; Millipore, Temecula, CA, USA); rabbit antimyeloperoxidase (1:200; A0398; Dako, Carpinteria, CA, USA); rabbit antinuclear factor-kappa B (p65) (1:200; sc-372; Santa Cruz Biotechnology, Santa Cruz, CA, USA); rabbit anticleaved caspase-3 (1:200; Asp175; Cell Signaling Technologies, Beverly, MA, USA); mouse antiglial fibrillary acidic protein (GFAP) (1:500; CY3 conjugated; C-9205; Sigma, St. Louis, MO, USA); goat antitumor necrosis factor-alpha (TNFα) (1:100; N-19, #SC-1350; Santa Cruz Biotechnology, Santa Cruz, CA, USA); rabbit anti-interleukin-1beta (IL-1β; #AB9787; Abcam, Cambridge MA, USA).

After several rinses in phosphate-buffered saline, the slides were incubated for 1 h with fluorescent-labeled species-appropriate secondary antibodies (1:500; Alexa Flour 488 and Alexa Flour 555; Invitrogen, Molecular Probes, Eugene, OR, USA) at room temperature. Omission of primary antibody was used as a negative control. The sections were coverslipped with polar mounting medium containing antifade reagent and 4′,6-diamidino-2-phenylindole (DAPI; Invitrogen, Eugene, OR, USA) and were examined using epifluorescence microscopy (Nikon Eclipse 90i; Nikon Instruments Inc., Melville, NY, USA). In some cases, for the sake of clarity of presentation, epifluorescence images are presented in the figures as “inverse” images, wherein the brightness value of each pixel is converted to the inverse value on a 256-step scale.

### Quantitative Immunohistochemistry

Quantifications comparing the effect of vehicle vs. heparin were made using the right side of the brain. Unbiased measurements of specific labeling within regions of interest (ROI) were obtained using NIS-Elements AR software (Nikon Instruments, Melville, NY, USA) from sections immunolabeled in a single batch, as previously described [26, 60]. All ROI images for a given signal were captured using uniform parameters of magnification, area, exposure, and gain. The following ROIs were defined: (1) for MPO, ED1, p65, TNFα, and IL-1β in the entorhinal cortex, a rectangular ROI, 1 × 1 mm, that was positioned on the pial surface; (2) for Iba1 in the hippocampus, a rectangular ROI that was positioned on the perforant pathway (see Fig. 6); (3) for Black Gold II in the hippocampus, a rectangular ROI that was positioned in the region between the perforant pathway and the dentate gyrus (see Fig. 7); (4) for Fluoro-Jade C in the hippocampus, a rectangular ROI, 450 × 360 μm, that was positioned at the hilus of the dentate gyrus. Segmentation analysis was performed by computing a histogram of pixel intensity for a particular ROI. Specific labeling was defined as pixels with signal intensity greater than two times that of background. For MPO, ED1, and p65, we performed object counts for specific labeling, which for MPO and ED1 corresponded to the number of cells. For Iba1 and Black Gold II, the area occupied by pixels with specific labeling was used to determine the percent area with specific labeling (%ROI). For Fluoro-Jade C, we counted the number of neurons within the ROI.

### Statistical Analysis

Calculations were performed with OriginPro8 (OriginLab Corp., Northampton, MA, USA). Statistical comparisons were made using Student’s *t* test or a one-way analysis of variance with Fisher’s post hoc comparison, as appropriate.

## Results

### SAH Involving the Entorhinal Cortex

**Fig. 1:**
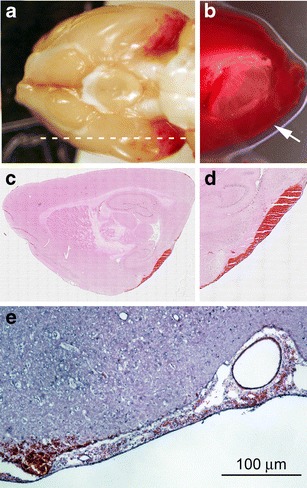
SAH involving the entorhinal cortex. **a** Image of the base of the brain showing SAH involving the entorhinal cortex bilaterally; *dotted line* location of parasagittal sections. **b** Parasagittal section of the brain stained with TTC showing the absence of infarction in the region of SAH (*arrow*). **c–e** Parasagittal sections of the brain in the region of SAH stained with H&E showing that the hemorrhage is localized largely in the subarachnoid space and that subarachnoid vessels show no evidence of vasoconstriction or vasospasm. All tissues were harvested 48 h after injury; the images shown are representative of findings in 28 rats (**a**), 3 rats (**b**), or 5 rats (**c**–**e**)

Using the methods described, we were able to take advantage of the natural curvature of the calvarium to reproducibly locate the subarachnoid space over the posterolateral temporal lobe and to produce a SAH that accumulated over the entorhinal cortex (Fig. 1a). The technique used, including the amount of blood and its slow injection, did not result in an ischemic injury to the region (Fig. 1b). Blood was largely confined to the subarachnoid space (Fig. 1c–e). Vasoconstriction or vasospasm of vessels in the subarachnoid space was not observed (Fig. 1e). This model was associated with near-zero mortality, with the rare deaths being attributable to anesthesia or surgery.

### Neurodegeneration in the Entorhinal Cortex

**Fig. 2:**
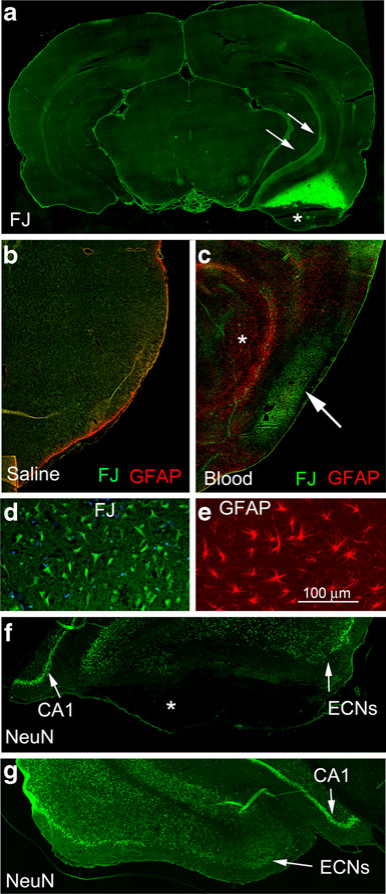
Cortical neurodegeneration following SAH. **a** Coronal section from a rat with unilateral injection of 50 μL of autologous blood into the subarachnoid space of the entorhinal cortex, stained with Fluoro-Jade C (*FJ*); note the bright staining above the background in the cortex and underlying white matter near the hemorrhage, along with the bright staining of the perforant pathway ipsilateral (*arrows*) but not contralateral to the hemorrhage; *asterisk* denotes hemorrhage. **b–e** Parasagittal sections of rat brains in the region of injection of saline (**b**) or blood (**c**–**e**), stained with Fluoro-Jade C (**b**–**d**) and immunolabeled for GFAP (**b**, **c**, **e**) showing degeneration involving neuron-like cells in the cortex beneath the SAH (**c**, **d**), but not with saline (**b**) and reactive astrocytes surrounding the region that stains for Fluoro-Jade C (**c**, **e**). **f**, **g** Coronal sections from a rat with unilateral injection of 50 μL of autologous blood into the subarachnoid space of the entorhinal cortex, immunolabeled for NeuN, showing the entorhinal cortex ipsilateral (**f**) and contralateral (**g**) to the hemorrhage; note the loss of ipsilateral entorhinal cortex neurons (*ECNs*) throughout the region, except laterally, and the preservation of neurons in the ipsilateral CA1 region and in all regions contralaterally (**g**); *asterisk* denotes hemorrhage; same rat as in **a**. All tissues were harvested 48 h after injury; the images shown are representative of findings in five rats (**b**–**e**) or three rats (**a**, **f**, **g**)

At 48 h after SAH, the adjacent cortex and white matter, including numerous neuron-like pyramidal cells, stained heavily with Fluoro-Jade C, as did the ipsilateral but not the contralateral perforant pathway (Fig. 2a, c, d). Saline injection did not result in staining with Fluor-Jade C above background levels (Fig. 2b). The cortical region that stained with Fluoro-Jade C showed downregulation of GFAP, but the surrounding tissues showed reactive astrocytosis (Fig. 2c, e). Compared to the control, the cortical region that stained with Fluoro-Jade C was depleted of NeuN-positive cells (Fig. 2f, g).

### Neuroinflammation in the Entorhinal Cortex

**Fig. 3:**
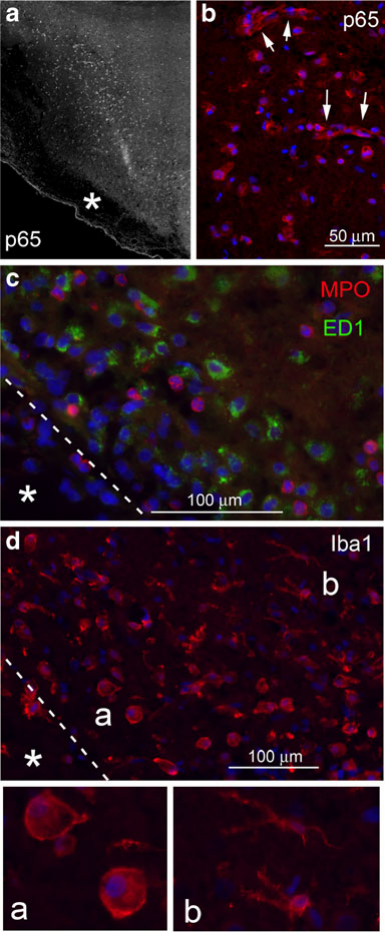
Cortical neuroinflammation following SAH. **a**, **b** Parasagittal section immunolabeled for NF-κB (p65), shown at low (**a**) and high (**b**) magnification, demonstrating (1) prominent expression of p65 subpially near the hemorrhage (*asterisk*) that tapers off with distance (**a**); (2) involvement of individual cells as well as elongated structures consistent with microvessels (*arrows*) (**b**); the image shown in **a** is an “inverse” epifluorescence image. **c** Parasagittal section immunolabeled for myeloperoxidase (MPO) to identify neutrophils (note also the polymorphic nuclei) and ED1 to identify macrophages and phagocytic, activated microglia, showing tissue infiltration beneath the pia (*dotted line*). **d** Parasagittal section immunolabeled for Iba1, to identify microglia, showing (1) large rounded cells (*a* and *inset a*) beneath the pia (*dotted line*), consistent with the phagocytic, activated microglia labeled with ED1 in **c**; (2) small cells with complex processes further away from the hemorrhage (*b* and *inset b*). All tissues were harvested 48 h after injury; the images shown are representative of findings in five rats

At 48 h after SAH, adjacent subpial tissues showed upregulation of the p65 subunit of nuclear factor-kappa B (NF-κB) in cells and microvessels, with p65 expression tapering off to normal within a few hundred micrometers (Fig. 3a, b). The subpial tissues were infiltrated with neutrophils and ED1-positive cells, consistent with macrophages and activated, phagocytic microglia (Fig. 3c). Immunolabeling for Iba1, which identifies all microglia regardless of their activation state, showed a gradient, from the pial edge to deeper, of cells with a rounded, activated morphology subpially that transitioned into a less activated morphology further away (Fig. 3d).

### Effect of Heparin on Neuroinflammation in the Entorhinal Cortex

**Fig. 4:**
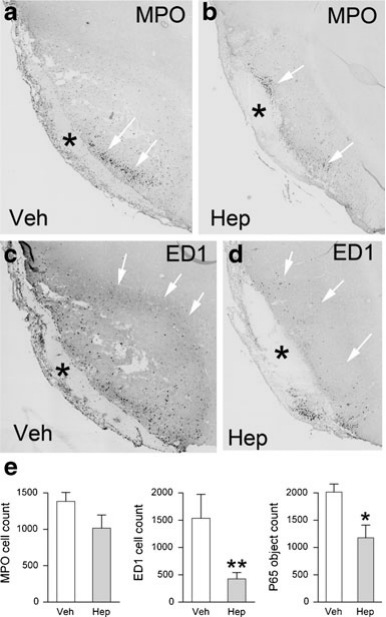
Heparin reduces cortical neuroinflammation (microglial activation) following SAH. **a–d** Parasagittal sections immunolabeled for myeloperoxidase (MPO) to identify neutrophils (**a**, **b**) or ED1 to identify macrophages and phagocytic, activated microglia (**c**, **d**) from rats treated with vehicle (**a**, **c**) or heparin (**b**, **d**); the advancing front of neutrophils, depicted by *arrows* (**a**, **b**), was similar without and with heparin; the advancing front of ED1-positive macrophages and microglia, depicted by *arrows* (**c**, **d**), was reduced by heparin; *asterisk* denotes the location of the SAH; the images shown are “inverse” epifluorescence images. **e** Bar graphs showing counts of MPO-positive cells, ED1-positive cells, and upregulation of p65 in rats treated with vehicle (*Veh*) or heparin (*Hep*); *P < 0.05; **P < 0.01; five rats per group

We examined the effect of heparin on counts of neutrophils, ED1-positive cells, and p65 upregulation in subpial tissues of the entorhinal cortex (Fig. 4). Immunolabeling for MPO showed that the subpial distribution of neutrophils appeared similar (Fig. 4a, b) and that counts of neutrophils were reduced, but not statistically different (Fig. 4e) in the two groups. Immunolabeling for ED1 showed that the “front” of ED1-positive cells appeared to extend less deeply (Fig. 4c, d) and that counts of ED1-positive cells were significantly reduced by heparin (Fig. 4e). Immunolabeling for p65 showed that upregulation of p65 was significantly reduced by heparin (Fig. 4e).

**Fig. 5:**
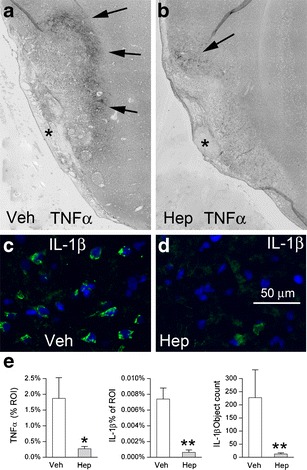
Heparin reduces cortical neuroinflammation (TNFα and IL-1β) following SAH. **a–d** Parasagittal sections immunolabeled for TNFα (**a**, **b**) or IL-1β (**c**, **d**) from rats treated with vehicle (**a**, **c**) or heparin (**b**, **d**); the advancing front of TNFα, depicted by *arrows* (**a**, **b**), was reduced by heparin; *asterisk* denotes the location of the SAH; the images shown in **a** and **b** are “inverse” epifluorescence images. **e** Bar graphs showing the percentage of the ROI immunolabeled for TNFα or IL-1β, as well as the number of cells immunolabeled for IL-1β, as indicated, in rats treated with vehicle (*Veh*) or heparin (*Hep*); *P < 0.05; **P < 0.01; five rats per group

We also examined the effect of heparin on the expression of TNFα and IL-1β in subpial tissues of the entorhinal cortex (Fig. 5). Immunolabeling for TNFα showed that the “front” of TNFα was less prominent and extended less deeply with heparin (Fig. 5a, b, e). Similarly, the expression of IL-1β as well as the count of IL-1β-positive cells were significantly reduced by heparin (Fig. 5c–e).

### Effect of Heparin on Neuroinflammation in the Hippocampus

**Fig. 6:**
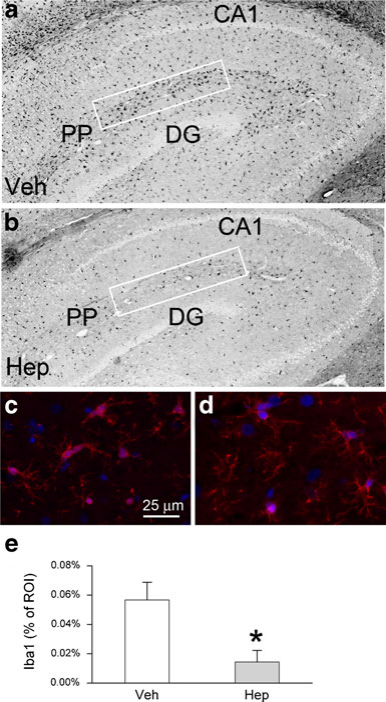
Heparin reduces hippocampal neuroinflammation following SAH. **a–d** Parasagittal sections immunolabeled for Iba1, shown at low (**a**, **b**) and high (**c**, **d**) magnification, from rats treated with vehicle (**a**, **c**) or heparin (**b**, **d**); note the “plump” morphology of microglia in the controls (**c**), which is absent with heparin (**d**); *CA1* cornu ammonis region 1, *PP* perforant pathway, *DG* dentate gurus; the ROI analyzed are depicted by *rectangles*; the images shown in **a** and **b** are “inverse” epifluorescence images. **e** Bar graph quantifying Iba1 in the ROI in rats treated with vehicle (*Veh*) or heparin (*Hep*); *P < 0.05; five rats per group

Immunolabeling for MPO, ED1, and p65 showed that none of these markers of inflammation were present in the hippocampus (not shown). Nevertheless, there was evidence of microglial activation, based on immunolabeling for Iba1 (Fig. 6), which identifies microglia regardless of activation state. In contrast to the Iba1-positive, ED1-positive, large, rounded, phagocytic microglia without processes observed subpially in the entorhinal cortex, microglial activation in the hippocampus was more muted. In the hippocampus of vehicle-treated rats, Iba1-positive cells were numerous (Fig. 6a); also, the cells were plump but not rounded and the processes were often enlarged (Fig. 6c). In heparin-treated rats, Iba1-positive cells were less prominent (Fig. 6b), the cell bodies were small, and the processes were finer (Fig. 6d). Upregulation of Iba1 was significantly reduced by heparin (Fig. 6e).

**Fig. 7:**
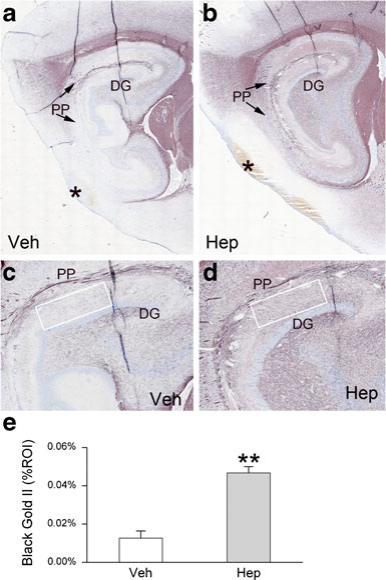
Heparin reduces demyelination of the perforant pathway following SAH. **a–d** Parasagittal sections stained with Black Gold II, shown at low (**a**, **b**) and high (**c**, **d**) magnification, from rats treated with vehicle (**a**, **c**) or heparin (**b**, **d**); *PP* perforant pathway, *DG* dentate gyrus; the ROI analyzed is depicted by the *rectangle*. **e** Bar graph quantifying Black Gold II-stained myelin in the ROI in rats treated with vehicle (*Veh*) or heparin (*Hep*); **P < 0.01; five rats per group

Myelin is highly susceptible to injury by oxidative stress [76]. We evaluated myelin preservation by staining with Black Gold II. Myelin was visibly deficient in the perforant pathway and in the dentate gyrus of vehicle-treated rats (Fig. 7a, c), consistent with the previous observation of Fluro-Jade C staining of the perforant pathway (Fig. 2a). By contrast, myelin appeared to be better preserved in the perforant pathway and dentate gyrus of heparin-treated rats (Fig. 7b, d). Quantification of myelin in the region between the perforant pathway and the dentate gyrus showed that myelin was significantly better preserved with heparin (Fig. 7e).

**Fig. 8:**
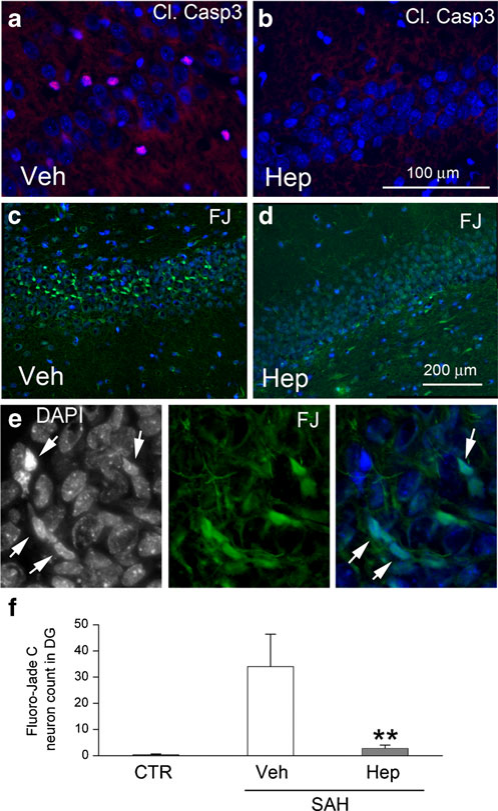
Heparin reduces apoptosis of granule cells of the dentate gyrus following SAH. **a–d** Parasagittal sections of the dentate gyrus immunolabeled for cleaved caspase-3 (**a**, **b**) or stained with Fluoro-Jade C (**c**, **d**) from rats treated with vehicle (**a**, **c**) or heparin (**b**, **d**). **e** Granule cells of the dentate gyrus from a vehicle-treated rat, double stained with DAPI and with Fluoro-Jade C (*FJ*), as indicated, showing pyknotic nuclei of apoptotic cells (*arrows*); superimposed images are also shown. **f** Bar graph quantifying Fluoro-Jade C-stained granule cells in the dentate gyrus of naïve (uninjured) rats (*CTR*), rats with bilateral SAH treated with vehicle (*Veh*) or heparin (*Hep*); **P < 0.01; three naïve rats and eight rats per group with SAH

In vehicle-treated rats, many granule cells of the dentate gyrus exhibited pyknotic nuclei that immunolabeled for cleaved caspase-3 (Fig. 8a) and that stained with Fluoro-Jade C (Fig. 8c). All Fluoro-Jade C-positive cells had pyknotic nuclei (Fig. 8e), also consistent with apoptosis. These findings were less prominent in rats treated with heparin (Fig. 8b, d). Quantification showed that Fluoro-Jade C-positive cells were significantly reduced with heparin (Fig. 8f).

## Discussion

We studied neuroinflammation in a model of SAH that involved the eloquent cortex and that was devoid of a global insult. To characterize microglial activation, we used two antibodies that convey complementary information. The ED1 antibody is a macrophage marker that recognizes an intracytoplasmic, lysosomal antigen whose expression increases during phagocytic activity in monocytes and other tissue macrophages, including microglia [3, 15, 28]. The Iba1 antibody is a marker directed against a microglia-specific antigen that identifies nonactivated (resting), as well as activated but nonphagocytic microglia that are ED1-negative [28]. Using these and other antibodies, we found a neuroinflammatory response in the entorhinal cortex adjacent to the hemorrhage that was robust, with invasion of neutrophils, ED1-positive cells, phagocytic microglia, and macrophages, and prominent labeling for NF-κB, TNFα, and IL-1β [16, 77]. This robust neuroinflammatory response was associated with significant neurodegeneration, marked by Fluoro-Jade C staining and loss of NeuN labeling. The robust inflammatory response in the entorhinal cortex was very different from the muted inflammatory response observed remotely in the hippocampus, where there was no labeling for MPO, ED1, or NF-κB and where the principal evidence for neuroinflammation was the appearance of Iba1-positive microglia that exhibited an activated morphology. Overall, our findings on neuroinflammation associated with SAH are consistent with previous reports [2, 11, 17, 24, 29, 52, 61, 64, 72, 73, 77, 78, 84]. What may be an importance difference, however, is that, in many of the previous reports, either the model used (endovascular puncture) or the amount of blood injected (200–500 μL) could potentially have been associated with a widespread or global ischemic insult, whereas in our experiments, the manner of injection and the amount of blood injected (50 μL on each side) were highly unlikely to produce an ischemic injury, as confirmed by the lack of vasoconstriction or vasospasm of vessels in the subarachnoid space and by uniformly positive tissue staining with TTC.

The major finding of the present study is that delayed IV infusion of heparin, at a dose that does not produce therapeutic anticoagulation, significantly reduced neuroinflammation associated with SAH, both the proximate effect in the entorhinal cortex, as well as the remote effect in the hippocampus. Heparin is a pleiotropic drug that has long been recognized to have broad anti-inflammatory and immunomodulatory activities that are independent of its anticoagulant effect [18, 44, 45, 65, 80, 86]. More than 100 heparin-binding proteins are known [86], with the growing list including numerous plasma proteins, proteins released from platelets, cytokines, chemokines, and other small, biologically active molecules, as well as endothelial cells themselves [14, 25, 32, 36, 51, 65]. The anti-inflammatory effects of heparin are due to a number of interactions: (1) inhibition of complement activation [23, 83]; (2) binding to the leukocyte adhesion molecules, P- and L-selectin [65, 81]; (3) inhibition of the cationic neutrophil proteases, human leukocyte elastase, and cathepsin G [23, 65]; (4) disruption of Mac-1 (CD11b/CD18)-mediated leukocyte adhesion to the receptor for advanced glycation end products (RAGE) [65]; (5) inhibition of RAGE ligation by its many proinflammatory ligands, including the advanced glycation end product, carboxymethyl lysine–bovine serum albumin, the nuclear protein, high mobility group box protein-1, and S100 calgranulins [46, 47, 53, 65, 67]; (6) inhibition of the nuclear translocation of NF-κB and a decrease in NF-κB–DNA binding in the endothelium [79].

A recent comprehensive review showed that heparin exhibits a broad diversity of biological effects, many of which can be directly linked to pathophysiological mechanisms that have been implicated in SAH-induced DNDs [74]. Among its wide-ranging effects, heparin (1) complexes with free hemoglobin, including oxyhemoglobin [1]; (2) blocks the activity of free radicals including reactive oxygen species [20]; (3) antagonizes endothelin-mediated vasoconstriction [12, 41, 85]; (4) accelerates antithrombin-III-mediated degradation of thrombin [57] which is implicated in protracted vasoconstriction [35]; (5) binds to several growth factors, thereby imparting antimitogenic [33, 34, 39] and antifibrotic [66] effects; and as reviewed above, (6) exerts potent multitargeted anti-inflammatory effects. The efficacy reported here for heparin against neuroinflammation, combined with the fact that the dose we used was less than that required for therapeutic anticoagulation [4, 42], suggests that heparin may be a safe prophylactic agent for reducing DNDs associated with SAH.

Despite the absence of a global insult, we identified extensive cell degeneration and cell death in this model, especially in the entorhinal cortex and white matter near the SAH. Hippocampal and cortical cell death and neuronal apoptosis have been previously documented, both in models of SAH and in humans with SAH [7, 54], but the trigger for apoptosis is not always known. With the endovascular perforation models, cell death has been attributed to a global insult, e.g., an elevation of the intracranial pressure resulting in global ischemia/hypoxia [19, 58, 59, 63]. However, several studies have shown that SAH produced by cautious blood injection can be also associated with neuronal apoptosis, independent of increased intracranial pressure [49, 50, 68, 82]. In the absence of a global ischemic insult, the actual trigger for cell death is often uncertain [7].

Given the absence of a global insult, the absence of direct mechanical or hemorrhagic injury to the hippocampus, and the muted inflammatory response in the hippocampus, we were initially surprised to find apoptosis of granule cells of the dentate gyrus. Apoptosis of granule cells was characterized by pyknotic nuclei that stained with Fluoro-Jade and showed cleaved caspase-3. The fact that we were injuring the entorhinal cortex gave rise to the possibility that, if not attributable to a global insult or to direct injury, apoptosis might be due instead to a transsynaptic process. Transsynaptic degeneration and transsynaptic apoptosis have been well documented under a variety of conditions in many brain regions following deafferentation, including the system of entorhinal cortex–perforant pathway–dentate gyrus that we studied here [27, 40, 71]. Our findings of degeneration/demyelination of the perforant pathway, as indicated by Fluoro-Jade C staining [69], and diminished labeling with Black Gold II are consistent with deafferentation of dentate gyrus cells secondary to the robust inflammatory response in the entorhinal cortex. Injury to the perforant pathway that causes deafferentation of granule cells renders these cells susceptible to excitotoxic injury and apoptotic death [40]. Moreover, transsynaptic apoptosis is worsened by neuroinflammation [13], as we observed here. Importantly, demyelination and transsynaptic apoptosis in our model were largely blocked by delayed IV infusion of low-dose heparin.

To our knowledge, transsynaptic apoptosis has not been previously identified in SAH. The potential implications of transsynaptic apoptosis in SAH may be large, especially if SAH involves the eloquent cortex. Distinct from generally recognized triggers of apoptosis, such as direct tissue trauma, ischemia, inflammation, etc., transsynaptic apoptosis involves the spread of cell death beyond the boundaries of the immediate injury to involve otherwise uninjured neurons that are an axon’s length away. This phenomenon may help to explain the prevalence of cognitive and neuropsychological deficits that commonly afflict humans with “mild-to-moderate” SAH who do not suffer a global insult [6, 10].

In summary, our findings here support previous work showing a robust neuroinflammatory response in the cortex underlying SAH which can lead to nearby tissue damage and cell death, as well as remote transsynaptic apoptosis. Our studies also show that delayed IV infusion of low-dose heparin exerts a powerful anti-inflammatory effect that lessens tissue injury and reduces transsynaptic apoptosis.
